# Cranio-Orbital and Orbitocranial Approaches to Orbital and Intracranial Disease: Eye-Opening Approaches for Neurosurgeons

**DOI:** 10.3389/fsurg.2020.00001

**Published:** 2020-02-07

**Authors:** Hussam Abou-Al-Shaar, Khaled M. Krisht, Michael A. Cohen, Abdullah M. Abunimer, Jayson A. Neil, Michael Karsy, Gmaan Alzhrani, William T. Couldwell

**Affiliations:** ^1^Department of Neurosurgery, Clinical Neurosciences Center, University of Utah, Salt Lake City, UT, United States; ^2^Department of Neurological Surgery, University of Pittsburgh Medical Center, Pittsburgh, PA, United States; ^3^Neurosurgery & Spine Associates, Montgomery, AL, United States; ^4^Department of Neurosurgery, Brigham and Women's Hospital, Harvard Medical School, Boston, MA, United States; ^5^Midwest Neurosurgery Associates, Kansas City, MO, United States; ^6^Department of Neurosurgery, National Neuroscience Institute, King Fahad Medical City, Riyadh, Saudi Arabia

**Keywords:** cranio-orbital, endoscopic, intraconal, intracranial, keyhole, total lateral orbitotomy, transconjunctival, zygoma

## Abstract

Orbital approaches for targeting intracranial, orbital, and infratemporal disease have evolved over the years in an effort to discover safe, reliable, effective, and cosmetically satisfying surgical corridors. The surgical goals of these approaches balance important factors such as proximity of the lesion to the optic nerve, the degree of anticipated manipulation and required space for surgical maneuverability, and the type of disease. The authors provide a comprehensive review of the most commonly used periorbital approaches in the management of intra- and extracranial disease, with emphasis on the advantages and limitations of each approach.

## Introduction

Orbitocranial surgical approaches involve collaboration between neurosurgeons and oculoplastic surgeons, each bringing a distinct, but partially overlapping, knowledge base to the treatment of disease. Anterior or middle fossa cranial skull base tumors can involve the orbit or orbital apex. The orbit is a small conical space comprising seven bones with complex relationships to the intracranial compartment. Exposure of the orbit is often performed during cranio-orbital approaches such as the orbitozygomatic or fronto-orbital craniotomy, where unroofing of the orbit can optimize the surgical view to the parasellar region. Neurosurgeons treating pathology in this location should become familiar with orbital anatomy and surgical approaches to this region. The difficulty in orbital approaches stems from the dense arrangement of eloquent neurovascular structures that funnel into the orbital apex and optic canal. Traditionally, neurosurgeons have exposed the superior and lateral surfaces of the orbit using frontotemporal, superior or lateral orbital approaches, whereas ophthalmologists exposed the orbit from the extracranial, transorbital approaches. Neurosurgeons are thus less familiar with transorbital approaches, while oculoplastic surgeons are less familiar with transcranial approaches to the orbit. In addition, as minimally invasive skull base approaches have gained popularity, several keyhole, endoscopic endonasal techniques and modifications to the traditional orbital routes have evolved to address lesions involving the intraorbital and intracranial compartments.

Orbitocranial approaches have been retooled from several routine ophthalmologic approaches to create new surgical corridors for neurosurgeons to access the intracranial compartment. Understanding the historical development of these approaches, their technical nuances, and their surgical limitations can aid surgeons in selecting the best approaches for their patients.

The goal of this paper is to review the most commonly employed approaches involving the orbit as used by neurosurgeons to address disease in the orbit, intracranial and extracranial compartments. The advantages and limitations of each approach will be discussed. A comprehensive understanding of differential diagnoses for orbital diseases is beyond the scope of this text, but a summary of diseases treated by these approaches can be seen in Tables 1, 2. The approaches can be generally divided into orbitocranial approaches that use orbital corridors to approach the intracranial compartment or cranio-orbital approaches that use intracranial corridors to approach the intraorbital compartment.

**Table 1:** Orbitocranial and cranio-orbital approaches: exposure, advantages, and disadvantages.

**Approach**	**Exposure**	**Advantages**	**Disadvantages**
**ORBITOCRANIAL APPROACHES**			
Lateral orbitotomy	• Lateral, superior, and inferior intraconal compartments • Orbital apex • Middle fossa and cavernous sinus	• Minimal orbitotomy for lateral orbital lesions • Wide exposure of orbit and orbital apex	• Enophthalmos
Total lateral orbitotomy	• Added exposure of anterior cranial fossa in addition to lateral orbitotomy exposure	• Exposes deep apex tumors • Deep seated orbital apex lesions obviating need for craniotomy	• Postoperative periorbital swelling • Cosmetic deformity • Enophthalmos
Modified lateral orbitotomy	• Sphenoid wing • Orbital apex • Middle fossa and cavernous sinus	• Good cosmetic outcome • Minimally invasive and enhanced recovery after surgery • Surgical exposure similar to pterional craniotomy but smaller opening	• Poor anterior cranial fossa exposure • Limited exposure for treating complex vascular lesions and tumors
Anterior medial micro-orbitotomy	• Medial intraconal compartment • Exposure medial to optic nerve	• Easy access to lesions medial to orbit and optic nerve • Better cosmetic outcome than orbitotomy	• Cannot address lesions at apex and superiorly located lesions • Endoscopic endonasal is useful alternative
Trans-conjunctival	• Inferomedial and lateral intraconal compartments • Can be used for supra-orbital keyhole exposure of anterior fossa	• Excellent cosmetic outcome • Low risk of enophthalmos	• Poor sphenoid wing, middle fossa, and orbital apex exposure
**CRANIO-ORBITAL APPROACHES**			
Pterional	• Versatile approach for superior and lateral orbital compartment and full exposure of orbital apex • Anterior and middle fossa	• Excellent exposure of orbital apex • No damage to intraorbital structures, less risk of enophthalmos • Incision behind hairline	• Requires craniotomy • Risk of temporalis atrophy
Mini-pterional	• Similar exposure as pterional	• Smaller incision behind temporal hair line	• Smaller working corridor • Poor orbital exposure with posterior hair line
Orbitozygomatic	• Removal of orbital roof and wall provides enhanced exposure of orbital apex and suprasellar region • Removal of zygoma provides enhanced exposure of middle fossa and infratemporal fossa	• Enhanced exposure • Less brain retraction	• Added operative time • Periorbital hematoma • Risk of enophthalmos
Lateral supraorbital	• Anterior cranial fossa • Superior orbital compartment	• Minimal disruption to temporalis muscle	• Large pterional incision behind hairline necessary to provide exposure
Supraorbital keyhole	• Anterior cranial fossa • Superior orbital compartment	• Minimally invasive approach through eyebrow incision	• Smaller craniotomy provides limited maneuverability of instruments • Cosmetic outcome affected by thickness of eyebrow
Endoscopic endonasal	• Medial orbital compartment • Medial aspect of orbital apex • Opticocarotid recess	• No visible scar • No orbitotomy or craniotomy • Low risk of enophthalmos	• Limited exposure of orbital apex • Increased risk of CSF leak and infection • Risk of infraorbital hypoesthesia

**Table 2:** Organization of differential diagnosis of orbital pathology.

**Intraconal**	**Extraconal**
**Optic nerve involvement**	**Intraorbital lesions**
Optic nerve glioma	Dermoid cyst
Optic nerve sheath meningioma	Lacrimal gland lesions
Optic neuritis	Dacryocystocele
Pseudotumour	Capillary hemangioma
Lymphoma/leukemia	Cavernous hemangioma
Intracranial hypertension	Lymphangioma
Retinoblastoma	Plexiform neurofibroma
**No optic nerve involvement**	Malignant peripheral nerve sheath tumors
Cavernous hemangioma	Orbital pseudotumor
Capillary hemangioma	Langerhans cell histiocytosis
Orbital varix	Rhabdomyosaroma
Arteriovenous malformation	Lymphoma/leukemia
Retro-orbital hematoma Lymphangioma	**Extraorbital lesions extending into extraconal space**
Schwannoma	Infections (cellulitis, sinusitis)
Sarcoidosis	Mucocele
Erdheim-Chester disease	Metastasis
Coloboma	Sinonasal tumors
Metastasis	Squamous cell carcinoma
Melanoma	Olfactory neuroblastoma
Carotid-cavernous fistula	Lymphoma
	Adenocarcinoma
	Adenoid cystic carcinoma
	Meningioma
	Hemangiopericytoma
	**Lacrimal gland lesions**
	Pleomorphic adenoma
	Adenoid cystic carcinoma
	Lymphoma/leukemia

## Orbitocranial Approaches

### Lateral Orbitotomy

**Figure 1:**
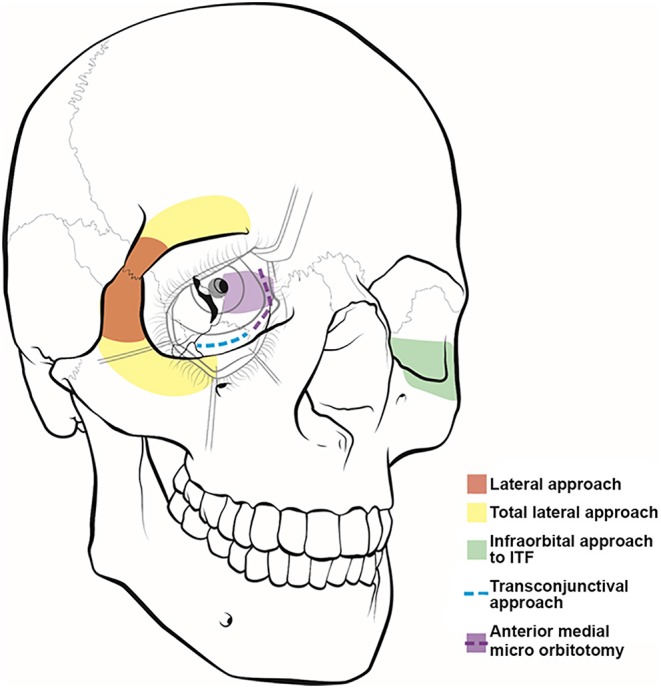
Illustration of orbitocranial approaches. These approaches include the lateral (red) and total lateral (yellow) approaches, the infraorbital approach to the infratemporal fossa (ITF) (green), the transconjunctival approach (blue), and the anterior medial micro-orbitotomy (purple).

An orbitotomy is an important surgical approach for resection of orbital tumors and accessing vascular pathologies. In 1889, Krönlein (1) first described the lateral orbitotomy, which provides excellent exposure of the temporal compartment of the orbit (Figure 1). It was indicated for periorbital and intraconal tumors located dorsally, basally, and laterally to the optic nerve (2–4). McNab and Wright (5) operated on 85 cases of cavernous hemangiomas, with a lateral orbitotomy used in 71 cases. The approach was highly efficacious, with only three patients experiencing visual loss postoperatively. Postoperative scar is one of the noted limitations of this procedure.

Several modifications have been proposed to the orbitotomy technique described by Kronlein (1). The Berke-Reese approach provides a more direct access to the orbit with a smaller, straight, horizontal cut made lateral to the orbit (6). Similarly, the Stallard-Wright lateral orbitotomy approach averts the lateral canthotomy through a curvilinear incision beginning above the eyebrow and slanting downward into the temporal area (7). Lately, surgeons have been performing lateral orbitotomies through upper lid crease incisions with limited canthotomy to minimize the cosmetic complications of this approach (8, 9). With these modifications as well as improvements in surgical technologies (e.g., drills and saws), this approach can be used more effectively and with better predicted cosmetic outcome.

**Figure 2:**
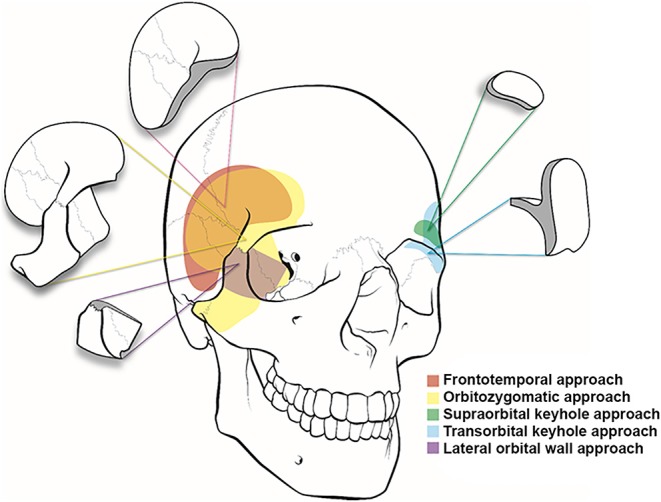
Illustration of cranio-orbital and orbitocranial approaches to the intracranial cavity. Frontotemporal or pterional (red), orbitozygomatic approach (yellow), supraorbital keyhole approach (green), transorbital keyhole approach (blue), and lateral orbital wall approach (purple).

Indications for a lateral orbital approach include tumors that involve the lateral orbit, orbital apex, middle fossa, and cavernous sinus (10). To obtain a surgical corridor, the surgeon starts by performing a lateral canthotomy, which is extended by cutting the superior and inferior crura of the lateral canthal tendon. Dissection is then carried out to the lateral orbital rim, where the periosteum is dissected by using a Freer periosteal elevator. The temporalis muscle is then elevated and mobilized to expose the lateral orbital wall. The periorbita is dissected from the intraorbital lateral orbital wall posterior to the zygomaticosphenoid suture. The zygomaticofacial and zygomatic-temporal arteries can be cauterized if encountered. The orbitotomy cuts are made at the frontozygomatic suture line superiorly and the body of the zygoma inferiorly (Figure 2). The orbital contents are protected during these cuts by using a malleable retractor or corneal shield. Once the orbitotomy is complete, the greater wing of the sphenoid can be drilled with a cutting bur to expose the orbital apex, superior orbital fissure, anterior clinoid process, and cavernous sinus. The lateral orbital rim can be replaced at the end of the surgery using low-profile titanium plates. Finally, skin closure is done after repairing the lateral cantholysis.

### Total Lateral Orbitotomy

The indications for a total lateral orbitotomy include extensive tumors involving the orbital apex, superior and inferior orbital fissures, and pterygopalatine fossa. The total lateral orbitotomy is similar to the lateral orbital approach, but the bone flap is extended to encompass the superior orbital rim up to the superior orbital notch and the body of the zygoma extending medially to the midpoint of the inferior orbital rim (Figure 1). The superior orbital rim is exposed by dissecting the retro-orbicularis oculi fat pad. By removing this rim, the surgeon will gain access to the anterior cranial fossa. The diploic space of the frontal bone serves as a landmark for identifying the entrance into the dural cavity, which lies within 11 mm below the orbital rim. Similarly, the inferior exposure can be facilitated with a subconjunctival, infraorbital incision. The superior ophthalmic vein, supraorbital artery, posterior ethmoid artery, and nasociliary nerve must be identified and preserved while performing this approach (11).

Extension of the lateral orbitotomy has been facilitated by the increased use of craniofacial fixation plates and improved surgical tools. Xiao et al. (12) introduced the concept of total lateral orbitotomy after analyzing the clinical results of several lateral bone flaps. The authors concluded that removing the superior and inferior orbital rims (Figure 1) would improve the surgical exposure and increase the rate of tumor resection. However, other studies have reported potential complications to this technique, including injury to the lateral rectus muscle, ciliary ganglion, short ciliary nerve, and optic nerve and its blood supply secondary to traction and orbital hemorrhage (11).

### Modified Lateral Orbitotomy

**Figure 3:**
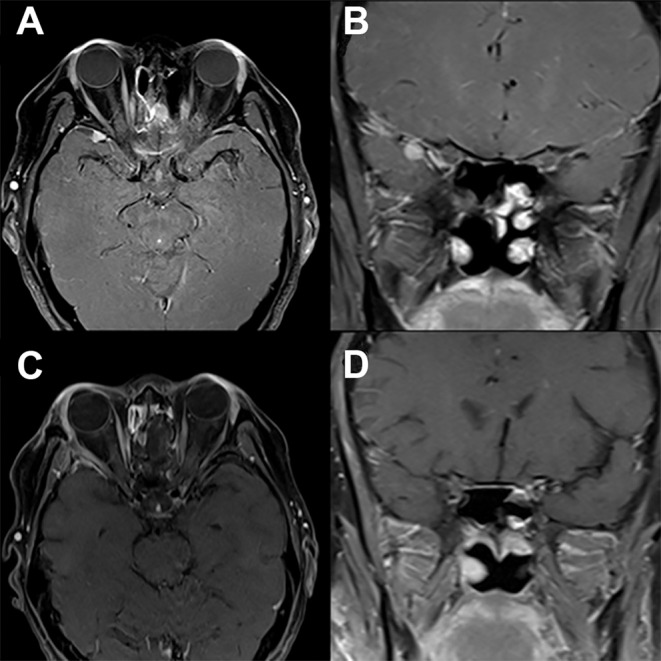
Modified lateral orbitotomy. Preoperative T1-weighted axial **(A)** and coronal **(B)** gadolinium-enhanced MRI in a patient with previous resection of esthesioneuroblastoma 5 years earlier demonstrate a new homogenously enhancing lesion along the right sphenoid wing. The location and small size of the lesion allows for complete removal via a right modified lateral orbitotomy orbitocranial approach. Postoperative T1-weighted axial **(C)** and coronal **(D)** gadolinium-enhanced MRI demonstrate complete resection of this lesion associated with full recovery and no new neurological findings.

The senior author (WTC) has proposed a novel minimally invasive, extradural, orbitocranial approach through the lateral orbital wall to access cavernous sinus disease (13). The modified lateral orbitotomy approach uses a classic transorbital incision, without incising the canthal ligaments, to open the lateral orbital rim and provide direct access to the sphenoid wing (Figure 3). This approach provides exposure of the primary working corridor of the pterional craniotomy, which is the sphenoidal segment of the Sylvian fissure, opticocarotid cistern, anterior clinoid process, and cavernous sinus. At our institution, we use this approach to obtain biopsy samples or resect small lesions in the anterior cavernous sinus. This approach could also be used to treat aneurysms or small brain tumors occurring in the parasellar region.

A lateral canthotomy incision is made, and the incision is forked along the superior eyelid to provide increased exposure. The lateral canthal ligaments should not be incised. Approximately 15 mm of lateral orbital rim is exposed where temporalis fascia and periorbita are dissected off the lateral and medial borders of the orbital rim, respectively. Two cuts are made in the lateral orbital rim by using a rotating drill or reciprocating saw: one inferiorly, just above the body of the zygoma, and a second superiorly, just below the frontozygomatic suture (Figure 2). The sphenoid wing can be drilled to expose the periorbita and temporal lobe dura if needed. The superior orbital fissure can be unroofed. Next, the temporal lobe dura is dissected off the lateral wall of the cavernous sinus, exposing the cavernous sinus and middle fossa structures. An extradural anterior clinoidectomy can be performed to expose the clinoidal segment of the internal carotid artery, the proximal and distal dural rings, and the anterior wall of the cavernous sinus. The lateral orbital rim is replaced with low-profile titanium plates at the completion of surgery. Patient follow-up demonstrates that complete removal of lesions (Figure 3) can be obtained via this approach with no new neurologic defects.

Limitations of this approach are similar to those of the supraorbital keyhole approach and include limited maneuverability and cosmetic outcome.

### Anterior Medial Micro-Orbitotomy

The first documented attempt for a medial orbital approach dates back to 1973, when Galbraith and Sullivan (14) described a surgical procedure for decompressing the perioptic meninges to relieve papilledema. The anterior medial micro-orbitotomy (Figure 1) is a minimally invasive approach to address medial orbital disease. Anterior and medial tumors confined within the periorbital intraconal region in the anterior two-thirds of the orbital cavity can often be accessed via this approach. This approach is extremely valuable in approaching small medial orbital tumors such as cavernous hemangiomas, orbital schwannomas, hemangiopericytomas, and neurofibromas (15, 16). A special self-retaining retractor has been designed to facilitate this operation. An eyelid speculum is inserted to protect the orbital contents, and a circumferential periectomy is performed around the cornea. Conjunctival relaxing incisions are made superior and inferior to the medial rectus muscle, and a muscle hook is placed to retract the muscle further medially. The lid speculum is then removed, and the specially designed medial orbital, self-retaining retractor is inserted. The handle of the retractor is angled so that it rests on the temporalis muscle laterally. An enucleation spoon may be used within the medial orbital compartment for further lateral retraction. Next, dissecting retractor blades are inserted to expose the medial orbit and the intraorbital fat for further dissection. Once the retractor is set in place, the operating microscope can be brought in. The dissection can then proceed deeper into the intraconal compartment.

Potential complications include injury to the medial rectus muscle or, less likely, the ophthalmic nerve. In addition, care must be taken when resecting the superior orbital masses because injury can occur to the supraorbital and trochlear nerves as they pass medially above the levator muscle. Hence, the endoscopic endonasal approach has become a popular minimally invasive alternative to address medially located orbital disease.

### Transconjunctival Approach

The transconjunctival approach (Figure 1) has been used in the treatment of intraorbital tumors located in the inferomedial, basal, and lateral regions of the orbit (5, 17). First, a lateral canthotomy incision is made, followed by lateral cantholysis. Next, the conjunctiva is incised in the inferior fornix and extended medially (Figure 1). The lower lid is divided into three regions: the preseptal region, which consists of the skin, subcutaneous tissue, orbicularis oculi muscle, and suborbicularis fascia; the septal region, which includes the orbital septum and tarsal plate; and the postseptal region, which contains the palpebral conjunctiva and the extension of the orbital fat. The postseptal route is preferred because the preseptal access may lead to ectropion as a result of septal damage (18). After the inferior orbital rim is exposed, the periorbita is incised to provide access to the intraconal compartment. The microscope is then brought into the operative field to allow for improved visualization of muscular and neurovascular structures (19).

This approach obviates the need for bone resection and subsequent reconstruction, making it a less traumatic approach with reduced incidence of enophthalmos. The approach also offers the advantage of producing no visible scar. Kiratli et al. (17) operated on 24 intraconal cavernous hemangiomas through the transconjunctival approach with satisfactory visual outcomes. Nevertheless, the transconjunctival approach is not indicated for medium- and large-sized tumors because of the limited surgical corridor. The potential complications of this approach include cicatricial entropion, ectropion of the eyelid, lower eyelid retraction, lateral telecanthus, lower eyelid detachment, canalicular laceration, chemosis, and lacrimal sac laceration (20).

## Cranio-Orbital Approaches

### Pterional Approach

Among the cranio-orbital approaches, the simplest is the frontotemporal or pterional craniotomy with preservation of the supraorbital rim proposed by Dandy (21) in 1922, which he adopted to access orbital apex tumors that could not be reached via a lateral approach. Subsequently, the approach went through further refinements and modifications (16, 22–25). Although this approach proves satisfactory in many instances, the additional removal of the supraciliary arch as proposed by Frazier (26) in 1913 for the treatment of pituitary tumors affords added working space and exposure with minimal brain retraction.

**Figure 4:**
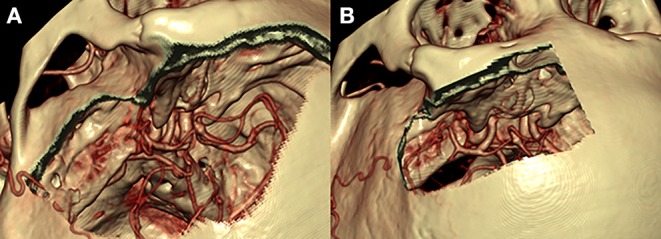
Comparison of the pterional **(A)** and supraorbital **(B)** craniotomies. The pterional craniotomy **(A)** provides excellent exposure of the anterior and middle cranial fossae with the center of exposure on the sylvian fissure and sphenoid wing. The supraorbital craniotomy **(B)** provides a more midline view of the anterior cranial fossa only with the center of exposure on the anterior fossa floor.

**Figure 5:**
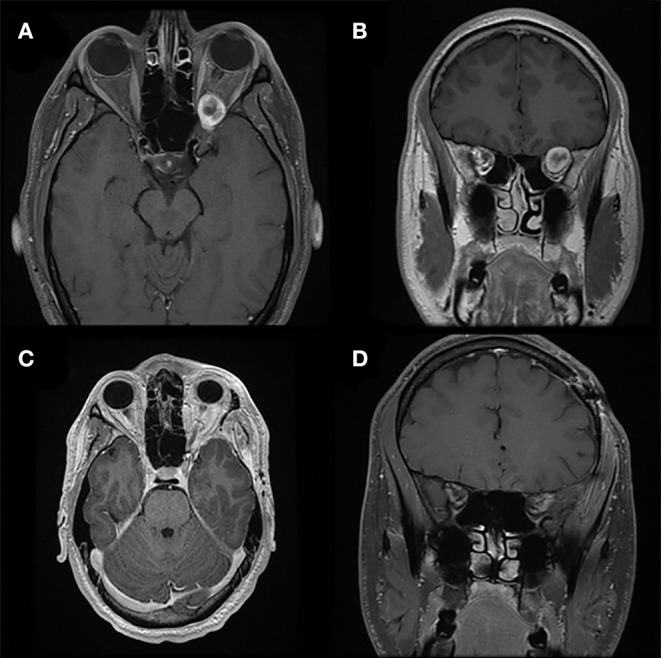
Pterional approach. Preoperative T1-weighted axial **(A)** and coronal **(B)** gadolinium-enhanced MRI of a patient with a heterogeneously enhancing lesion of the left orbital apex causing compression of the optic nerve and nerves exiting the superior orbital fissure. The location and larger size of the lesion requires a pterional cranio-orbital approach by a multidisciplinary team to remove the sphenoid wing and expose the superior and lateral aspects of the orbit and orbital apex. Postoperative T1-weighted axial **(C)** and coronal **(D)** gadolinium-enhanced MRI demonstrate complete resection of this lesion associated with resolution of symptoms.

The pterional approach provides an excellent view of the superior and lateral aspects of the posterior orbit, the optic canal, the superior orbital fissure, and the anterior temporal fossa and a panoramic view of the orbital apex that cannot be obtained using other orbital or orbitocranial approaches (Figures 2, 4). It is ultimately centered on the Sylvian fissure and provides access to the sphenoid ridge. Deep cranio-orbital tumors with intradural invasion can also be addressed via this approach (Figure 5). The excellent exposure of the pterional approach is primarily obtained by removing the sphenoid wing to expose the superior orbital fissure as well as the posterior and lateral orbit after the frontotemporal craniotomy (27). Interestingly, lesions at the posterior-medial portion of the orbit inferior to the optic nerve may be approached using a contralateral pterional craniotomy to view the medial aspect of the contralateral orbital apex (28).

The scalp incision begins at the level of the zygomatic arch (1 cm anterior to the tragus), proceeds up to the superior temporal line, and curves anteriorly and medially, ending in the midline at the hairline. Dissection is performed through the subgaleal plane where the musculocutaneous flap is then reflected inferiorly to reach the superior orbital rim, the temporal muscle flap is detached from the temporal fossa, and the lateral orbital wall is exposed. The craniotomy is started with drilling the MacCarty keyhole, where the periorbita, frontal lobe, and temporal lobe dura can be simultaneously exposed. A craniotome is used to create a frontotemporal bone flap centered on the sphenoid wing that typically exposes the anterior and middle cranial fossa floors and temporal tip. Removal of the sphenoid wing is facilitated using a cutting bur and rongeurs, until the meningo-orbital band (a dural band that connects the frontal and temporal dura with the superior orbital fissure) is reached. The meningo-orbital band signifies the transition from sphenoid wing to anterior clinoid process. The orbit can be exposed by continuous drilling of the sphenoid wing until the lateral orbital wall and orbital roof are encountered. Once the bone is thinned, a Kerrison punch can be used to unroof the periorbita. The orbit can be opened and intraorbital disease can be addressed by working through the superior and lateral rectus muscle corridors. The roof of the optic canal can be drilled with a diamond bit, and circumferential decompression is accomplished with an anterior clinoidectomy and removal of the optic strut. The optic nerve and neurovascular contents of the superior orbital fissure can be followed through the orbital apex (annulus of Zinn) and into the orbit.

The pterional craniotomy requires a relatively large skin incision and extensive mobilization and partial devascularization of the temporalis muscle. The most common cosmetic problem after pterional craniotomy is the presence of depressed deformities in the pterional region due to temporalis muscle atrophy (29). Exposure of the orbit through the pterional approach often leads to a postoperative self-limiting intraorbital hematoma and periorbital edema; however, excellent outcomes with complete resection can be achieved with this approach (Figure 5).

### Mini-Pterional Approach

The mini-pterional approach was first described by Figueiredo et al. (30) as a less-invasive modification of the standard pterional craniotomy. The arcuate scalp incision is centered on the sphenoid ridge, beginning about 1 cm above the zygomatic arch and ending at the mid-pupillary line. The mini-pterional approach was found to be comparable to the pterional approach in a quantitative analysis of surgical exposure (30). Caplan et al. (31) and Sabersky et al. (32) published their experiences with the mini-pterional approach to treat a total of 129 patients with unruptured aneurysms of the middle cerebral artery and internal carotid artery and found adequate exposure in all cases and improved cosmetic outcome.

Although the mini-pterional approach provides comparable exposure to the pterional craniotomy, there are several limitations of this approach. The smaller craniotomy size may limit the space for frontal and temporal lobe mobilization for larger lesions. Exposure of the orbit is fairly similar to the pterional approach, although the exposure at the medial orbital roof and inferolateral orbital wall may be limited. Patients with posterior temporal hairlines or with increased scalp tension may not provide adequate anterior exposure of the lateral orbital rim using the mini-pterional incision, which could lead to decreased exposure. The mini-pterional approach is a useful less-invasive alternative to the pterional craniotomy for properly selected patients.

### Orbitozygomatic Approach

As described by Pellerin et al. (33) and Hakuba et al. (34), the anterior and middle cranial fossae, basilar apex region, and upper clivus can be optimally visualized using the orbitozygomatic approach (Figure 2). Compared with the pterional craniotomy, the orbitozygomatic approach provides similar exposure of the orbit and orbital apex. By removing the zygoma, orbital rim, and entire orbital roof and lateral wall, the orbitozygomatic approach provides improved operative trajectory with multidirectional access and less brain retraction through greater bone removal at the skull base and mobilization of the orbital contents.

This extensive approach (Figure 2) provides access to disease in the orbital apex, basilar apex, paraclinoid and parasellar regions, cavernous sinus, and anterior and middle fossa floors (35). As in the pterional approach, the scalp incision begins at the inferior border of the zygomatic arch (1 cm anterior to the tragus), arching upward and anteriorly, and ends at the anterior-most aspect of the hairline in the midline. Often, the incision can be extended just behind the hairline to the contralateral mid-pupillary line to provide additional orbital exposure. To preserve the facial nerve, which lies approximately 2 cm below the zygomatic arch over the mandibular condyle, the incision should not be extended inferiorly below the zygomatic arch. To complete the exposure, the temporal muscle is incised and elevated. Avoidance of monopolar cauterization can help minimize devascularization of the temporal muscle and risk of muscle atrophy. It is also important to note that extensive dissection of the subgaleal fat pad should be avoided to minimize the risk of injuring the frontal branch of the facial nerve, which runs within the superficial temporalis fascia just above the fat pad. Subfascial or interfascial dissection can be used to extend the exposure further toward the orbit while lessening the risk of injury to the frontal branch of the facial nerve. The dissection proceeds until the desired length of zygoma, lateral orbital wall, and superior orbital rim are exposed. Additionally, the periorbita is dissected and freed from its attachments with the superior and lateral aspects of the intraorbital roof and lateral wall.

A two-piece orbitozygomatic craniotomy is performed by first performing a standard pterional craniotomy. After that, the orbitozygomatic bone flap is removed in one piece using 6 bone cuts, as described by Zabramski et al. (36). The addition of orbital rim exposure or detachment of the zygomatic arch aids in the exposure of the superior and lateral orbit, respectively. As the orbitozygomatic bone flap is removed, any soft tissue attachment should be carefully freed. At the end of surgery, both the orbitozygomatic and pterional bone flaps are fixed in position with no further bone reconstruction required.

Alternatively, a one-piece orbitozygomatic or modified orbitozygomatic craniotomy can be performed. The surgical steps are similar to the aforementioned technique except that the pterional bone flap is not freed because drilling stops at the orbital rim just lateral to the supraorbital notch and at the pterion from below (35). To avoid driving the orbital roof into the frontal lobe, the 1-piece bone flap should be removed in a medial-to-lateral direction. At the end of surgery, the bone flap is fixed with miniplates and screws.

The approach has been applied effectively with excellent outcomes for resection of various neoplastic and vascular lesions (37, 38). Nevertheless, potential complications are associated with this additional exposure, including increased risk of injury to the frontal branch of the facial nerve (39). Because of the inherent limitations of the pterional and orbitozygomatic approaches and the desire to offer less-invasive treatment options, surgeons started employing more minimally invasive cranio-orbital approaches such as the supraorbital keyhole approach, as well as orbitocranial approaches such as the modified lateral orbitotomy, which provide similar exposure as the pterional/orbitozygomatic craniotomy through a smaller working corridor with superior aesthetic outcomes.

### Lateral Supraorbital Approach

Several approaches centered on the supraorbital rim have been reported, including the supraorbital and supraorbital keyhole approaches (40–42). Jane et al. (43) first introduced the supraorbital approach in 1982 to treat parasellar and frontal skull base lesions. The supraorbital approach provides an excellent view of the suprasellar anatomy in an anterior-to-posterior trajectory, eliminating barriers such as the temporal lobe and sphenoid wing. The supraorbital approach enables the surgeon to address pathologies located in the intraorbital, intracranial-extradural, and subarachnoid spaces. Unlike the pterional approach centered at the Sylvian fissure and sphenoid wing with access to the anterior and middle cranial fossae, the supraorbital approach provides access to the anterior cranial fossa and can be used to treat anterior fossa tumors and anterior circulation aneurysms (Figures 2, 4). Traditionally, a standard pterional skin incision is performed to provide supraorbital frontal bone exposure with minimal disruption in the temporalis muscle. Next, a frontal burr hole is drilled in the pterional region, and a limited frontal craniotomy is performed flush with the anterior cranial fossa floor. The surgical field can be extended into the periorbita by removing the superior orbital rim and the orbital roof. The microscope is then brought to the field, and the microsurgical corridor is dissected down to the targeted pathological tissue.

The supraorbital approach is a variant of the pterional approach with no temporal bone removed and provides more limited access to the anterior cranial fossa and superior aspect of the orbit. Limitations include risk of injury to the frontalis branch of the facial nerve, as a galeocutaneous flap is typically reflected and the temporalis muscle is left mostly attached to the temporal bone. This risk can be mitigated using subfascial or interfascial dissection as previously discussed.

### Supraorbital Keyhole Approach

In 1998, Perneczky et al. (44) were the first to advocate the keyhole concept in neurosurgery; their series included 1297 cases of cerebral aneurysms and 221 tumor cases using the supraorbital keyhole approach with an incision within the eyebrow (Figure 2) (45). For cosmetic purposes, a small skin incision is placed through the lateral two-thirds of the eyebrow along the superior orbital rim to avoid the supraorbital nerve medially and the frontal branch of the facial nerve superiorly. A meticulous dissection of the orbicular muscle with minimal retraction is required to prevent a postoperative periorbital hematoma. The burr hole is drilled in the pterional region, and a small supraorbital craniotomy is drilled. The same microsurgical technique and nuances are used through a much smaller opening. Cheng et al. (46) quantitatively demonstrated that the area of the parasellar region accessed through the smaller keyhole supraorbital approach is as adequate as its larger supraorbital and pterional counterparts. An endoscope can be used to expand the surgical view using the keyhole approach. The minimal soft tissue dissection and small craniotomy size reduce postoperative orbital and frontotemporal swelling and may lead to faster recovery. The approach is generally safe and effective in managing various neoplastic and vascular entities. In the series by van Lindert et al. (47), there were no approach-related complications encountered in 139 supraorbital keyhole approaches performed for 197 supratentorial aneurysms.

The main limitations of the keyhole supraorbital approach include decreased surgical maneuverability, potential scar formation in the eyebrow, supraorbital numbness, and the possibility of frontal sinus infection and meningitis, if a breached frontal sinus is not obliterated.

### Extended Transethmoidal, Transsphenoidal, and Transmaxillary Approaches

**Figure 6:**
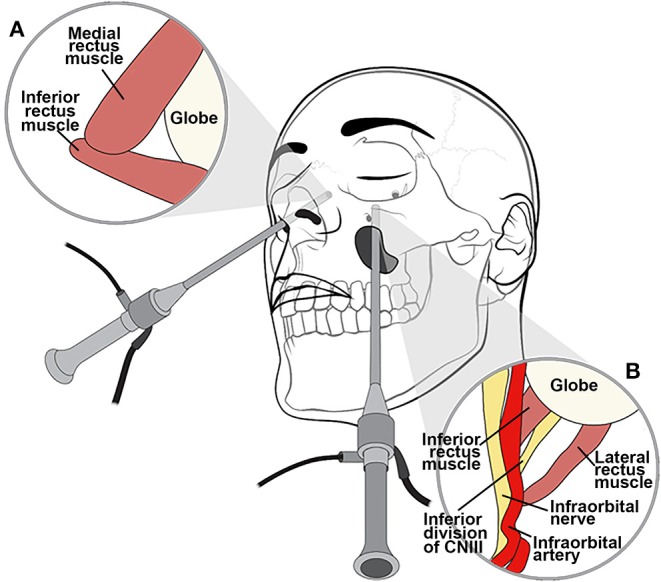
Illustration of extended endoscopic endonasal approach. An endoscopic endonasal approach through the medial orbital wall relies on the medial and inferior rectus muscles, which are positioned lateral to the ethmoid lamina papyracea as seen from a contralateral approach **(A)**. The transantral endoscopic approach uses the natural workable space of the maxillary sinus to access the floor of the orbit relying on the inferior and lateral rectus muscles for orientation with respect to the optic nerve **(B)**.

Endoscopic endonasal approaches have revolutionized and changed the management paradigm for many anterior skull base and orbital lesions that were deemed inoperable before or were associated with significant complications (Figure 6). The approach provides a cosmetically superior alternative to traditional medial orbital and orbitocranial approaches to the orbit, along with transcranial and cranio-orbital approaches to the anterior skull base and orbital apex.

The endoscopic endonasal trans-ethmoidal approach is an excellent approach for tumors located inferior and medial to the optic nerve (48, 49). Similarly, the endoscopic endonasal transsphenoidal approach provides access to the medial aspect of the orbital apex, where the intracranial and intraorbital optic nerve can be decompressed. Via these approaches, the medial and inferior orbital structures and orbital apex can be accessed with no skin incision required. Additionally, minimal neurovascular manipulation and no brain retraction are needed during surgery.

**Figure 7:**
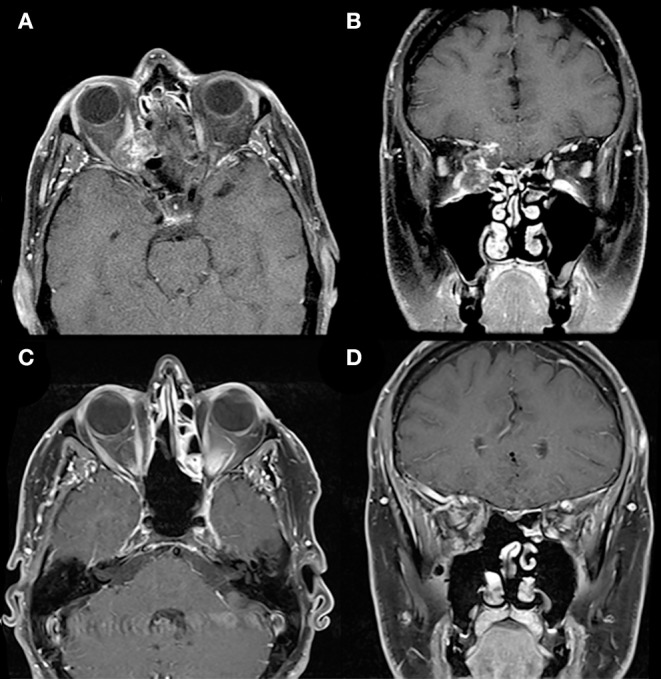
Extended endoscopic endonasal approach. Preoperative T1-weighted axial **(A)** and coronal **(B)** gadolinium-enhanced MRI demonstrate a right heterogeneously enhancing lesion involving the medial orbit, eroding through the lamina papyracea medially and the anterior skull base superiorly, but respecting the maxillary sinus inferiorly. The lesion compresses the right orbital contents including the optic nerve. The medial location of the lesion and erosion into the ethmoid sinus facilitated an endoscopic endonasal transmaxillary transorbital approach by a multidisciplinary team. Postoperative T1-weighted axial **(C)** and coronal **(D)** gadolinium-enhanced MRI demonstrate complete resection of this lesion.

Endoscopic endonasal approaches are useful for medial orbital disease with or without nasal sinus involvement (Figure 7). Additionally, these approaches are effective in managing various pathologies involving the orbital apex and paraclinoid region. Endoscopically, the middle turbinate is excised on the ipsilateral side of interest to widen the nasal corridor. Subsequently, uncinectomy and middle meatal antrostomy are performed. An anterior ethmoidectomy is performed, and the anterior ethmoidal artery is identified immediately posterior to the frontal recess. Next, a posterior ethmoidectomy is performed to identify the posterior ethmoidal artery in the superior portion of the lamina papyracea. The anterior and posterior ethmoidal arteries should be preserved unless attempting to devascularize an anterior skull base tumor. A posterior septectomy is completed after fracturing the septum off the sphenoid rostrum. A wide sphenoidotomy is performed bilaterally, exposing the opticocarotid recess. The periorbita can be identified after the lamina papyracea and medial orbital wall are removed. Using an angled endoscope, two important landmarks are identified for the endoscopic endonasal transorbital approach: the medial rectus muscle, found lateral to the lamina papyracea, and the inferior rectus muscle, found superior to the orbital floor (Figure 6). The space between the medial and inferior rectus muscles provides a surgical corridor to approach the optic nerve and orbital apex. It is important to note that varying degrees of posterior ethmoid development and anterior clinoid pneumatization might affect the position of the orbital apex.

For inferolateral pathology, the endoscopic transmaxillary approach could be considered. The transmaxillary endoscopic approach uses the natural workable space of the maxillary sinus to create direct access to the floor of the orbit, relying on the inferior and lateral rectus muscles for orientation with respect to the optic nerve, which is found more medially (Figure 6). This working corridor may be used to penetrate deeper into the retrobulbar space and may prove to be an attractive alternative to the standard lateral orbitotomy.

This less-invasive surgical technique provides excellent magnification and illumination of the viewed structures with the aid of angled vision to acquire comprehensive assessment of the surgical field. Nevertheless, the endoscopic technique involves a few limitations, such as the steep angle required to reach the lateral orbit, the lack of binocular vision, and the subsequent lack of depth perception. One of the major complications of using this approach is cerebrospinal fluid leak, which can be prevented by using a vascularized nasoseptal flap, along with other adjunct skull base reconstruction techniques. Despite these limitations, complete tumor removal can be achieved (Figure 7).

## Conclusions

Cranio-orbital, orbitocranial, and endoscopic endonasal approaches can be used to access lesions of the anterior skull base and orbit with excellent outcomes and minimal morbidity rates. Minimally invasive cranio-orbital and orbitocranial approaches should be used judiciously, with consideration of the pathology being treated and the degree of orbital and cranial exposure necessary. Although the orbital anatomy is complex and unfamiliar to many surgeons, knowledge of these techniques and their nuances is essential for dealing with orbital and anterior cranial fossa disease.

## Author Contributions

HA-A-S was involved in conception of the review, acquisition and interpretation of information, and drafting and revising the manuscript. KK was involved in conception of the review, acquisition and interpretation of information, and drafting and revising the manuscript. MC was involved in acquisition and interpretation of information and drafting and revising the manuscript. AA was involved in drafting and revising the manuscript. JN was involved in acquisition and interpretation of information and revising the manuscript. MK was involved in acquisition and interpretation of information and drafting and revising the manuscript. GA was involved in acquisition and interpretation of information and revising the manuscript. WC was involved in conception of the review, revising the manuscript, and overall supervision.

### Conflict of Interest

The authors declare that the research was conducted in the absence of any commercial or financial relationships that could be construed as a potential conflict of interest.
